# The redox metabolic pathways function to limit *Anaplasma phagocytophilum* infection and multiplication while preserving fitness in tick vector cells

**DOI:** 10.1038/s41598-019-49766-x

**Published:** 2019-09-13

**Authors:** Pilar Alberdi, Alejandro Cabezas-Cruz, Pedro Espinosa Prados, Margarita Villar Rayo, Sara Artigas-Jerónimo, José de la Fuente

**Affiliations:** 1 SaBio. Instituto de Investigación en Recursos Cinegéticos IREC (CSIC-UCLM-JCCM), 13005 Ciudad Real, Spain; 2 UMR BIPAR, INRA, ANSES, Ecole Nationale Vétérinaire d’Alfort, Université Paris-Est, Maisons-Alfort, 94700 France; 3 Department of Veterinary Pathobiology, Center for Veterinary Health Sciences, Oklahoma State University, Stillwater, OK 74078 USA

**Keywords:** Pathogens, Mass spectrometry, Transcriptomics

## Abstract

Aerobic organisms evolved conserved mechanisms controlling the generation of reactive oxygen species (ROS) to maintain redox homeostasis signaling and modulate signal transduction, gene expression and cellular functional responses under physiological conditions. The production of ROS by mitochondria is essential in the oxidative stress associated with different pathologies and in response to pathogen infection. *Anaplasma phagocytophilum* is an intracellular pathogen transmitted by *Ixodes scapularis* ticks and causing human granulocytic anaplasmosis. Bacteria multiply in vertebrate neutrophils and infect first tick midgut cells and subsequently hemocytes and salivary glands from where transmission occurs. Previous results demonstrated that *A*. *phagocytophilum* does not induce the production of ROS as part of its survival strategy in human neutrophils. However, little is known about the role of ROS during pathogen infection in ticks. In this study, the role of tick oxidative stress during *A*. *phagocytophilum* infection was characterized through the function of different pathways involved in ROS production. The results showed that tick cells increase mitochondrial ROS production to limit *A*. *phagocytophilum* infection, while pathogen inhibits alternative ROS production pathways and apoptosis to preserve cell fitness and facilitate infection. The inhibition of NADPH oxidase-mediated ROS production by pathogen infection appears to occur in both neutrophils and tick cells, thus supporting that *A*. *phagocytophilum* uses common mechanisms for infection of ticks and vertebrate hosts. However, differences in ROS response to *A*. *phagocytophilum* infection between human and tick cells may reflect host-specific cell tropism that evolved during pathogen life cycle.

## Introduction

During evolution, aerobic organisms developed conserved mechanisms controlling the production of reactive oxygen species (ROS) to maintain redox homeostasis signaling and modulate signal transduction, gene expression and cellular functional responses under physiological conditions^1–3^. The production of ROS by mitochondria is essential in the oxidative stress associated with different pathologies and in response to pathogen infection^4–8^. The proximal mitochondrial (mt) ROS is superoxide (O_2_^.−^), which is predominantly produced by complex I (nicotinamide adenine dinucleotide (NAD)H/ubiquinone oxidoreductase) and complex III (ubiquinol/cytochrome c oxidoreductase), and causes mt dysfunction and apoptosis/necrosis^1,4^. The O_2_^.−^ is usually converted by mt superoxide dismutase (SOD 2) and GTPase Rac1/2 into H_2_O_2_, which can reach the cytoplasm after crossing mt membranes^1^. Other mt enzymes such as various isoforms of the cytochrome P450 superfamily could also generate ROS^1,9,10^. The other most relevant intracellular sources of ROS are NADPH oxidase and 5-lipoxygenase^1^. The host response to protect tissues from ROS rely on antioxidant defenses, which include mt catalase peroxisomes (CAT), glutathione peroxidase (GPX), Cu/Zn SOD, thioredoxin reductase (Trx-Red) and glutaredoxin (Glrx)^1^. Finally, redox sensors in eukaryotes affect different biological processes such as those regulated by the nuclear factor (NF)-κB, which controls mammalian mt dynamics^1,11^. In mammals, cytokines such as interleukin-1 (IL-1) and tumor necrosis factor (TNF) cause the activation of NF-κB and the production of intracellular ROS^1,11^.

Current knowledge supports that low levels of ROS result in antioxidant response required for normal cell homeostasis, while intermediate ROS levels activate NF-kB mediated pro-inflammatory, adaptive and anti-apoptotic responses for cell adaptation to stress and survival^1,5^. However, high levels of ROS cause the induction of apoptosis, irreversible cell injury and dead^1,5^. The production of ROS in response to pathogen infection could also have different outcomes^7^. ROS causes oxidative damage to biocompounds that can kill pathogens directly or indirectly stimulates pathogen elimination by various nonoxidative mechanisms^7^. The indirect nonoxidative mechanisms include pattern recognition receptors signaling, autophagy, neutrophil extracellular trap formation, and T-lymphocyte responses^7^. However, increasing evidences support that for certain bacterial pathogens such as *Mycobacterium abscessus* and *Helicobacter pylori*, ROS production increases pathogen burden through mechanisms that include the metabolic effects of ROS on pathogen physiology, ROS-induced damage to the immune system, and ROS-induced activation of immune defense mechanisms^7^.These mechanisms are hijacked by particular pathogens to balance effective microbicidal mechanisms of the immune system^7^.

Arthropod vectors transmit pathogens that affect human and animal health worldwide. The tick *Ixodes scapularis* is a vector of *Borrelia burgdorferi* and *Anaplasma phagocytophilum* in North America^12^. The infection and colonization of ticks by *A*. *phagocytophilum*, an obligate intracellular bacterium (Rickettsiales: Anaplasmataceae), occurs first in midgut cells and subsequently in hemocytes and salivary glands from where transmission occurs during feeding^13^. To establish infection, *A*. *phagocythophilum* induces complex cellular changes mediated mainly by transcriptional reprogramming and proteome modulation. These mechanisms appear to be common to tick and vertebrate hosts, and include but are not limited to manipulation of the immune response, inhibition of cell apoptosis, remodeling of the cytoskeleton, and modification of cell epigenetics and metabolism^14–18^. In mammals, *A*. *phagocytophilum* infects neutrophils and must modulate granulocyte major defenses such as the oxidative response^13^.

Previous results demonstrated that *A*. *phagocytophilum* does not induce the production of ROS as part of its survival strategy in neutrophils^19–21^. However, this bacterium induces the production of ROS in macrophages^22^, which is presumably why these cells are not suitable hosts^13^. Nevertheless, although *A*. *phagocytophilum* does not suppress a global respiratory burst in neutrophils, it significantly reduces NADPH oxidase subunits gp91(phox) and p22(phox) levels in its phagosome membrane^21^. The inhibition of ROS production in *A*. *phagocytophilum*-infected human promyelocytic leukemia HL-60 cells has been proposed to be regulated by pathogen effector Ankirin A (AnkA)-dependent down-regulation of NADPH oxidase^23^.

Obligate blood-sucking ectoparasites such as ticks respond to the oxidative stress and neutralize the ROS generated from blood meal digestion to avoid tissue damage using different antioxidant defense mechanisms^24–26^. Inorganic polyphosphates regulate the generation of ROS during embryogenesis in *Rhipicephalus microplus* ticks by increasing the activity of superoxide dismutase, catalase and glutathione reductase, providing evidence for the function of different enzymes in mt ROS metabolism in tick embryos^27^. The role of tick mt antioxidant defense proteins in blood feeding and reproduction has also been characterized in ticks^28,29^. However, little is known about the role of ROS during pathogen infection in ticks. Recently, Kalil *et al*.^30^ suggested that while *R*. *microplus* tick cells respond to microbial stimuli by increasing ROS production, the infection with *A*. *marginale* induces a decrease in ROS levels and upregulation of antioxidant responses. *Anaplasma phagocytophilum* infection induces the reduction of heme-responsive gene 1 (HRG1) protein levels, suggesting a mechanism to reduce heme release into the cytoplasm of midgut cells^31^. This mechanism appears to be manipulated by *A*. *phagocytophilum* to reduce the antimicrobial oxidative stress caused by ROS generated after heme release^31^. Furthermore, recent evidence suggests that *A*. *phagocytophilum* manipulates tick biological processes in order to facilitate infection, while ticks respond by limiting pathogen infection^15,32^. The resulting tick-pathogen association preserves feeding fitness and vector competence for survival of both ticks and pathogens^15,32^. The mechanisms used by *A*. *phagocytophilum* to manipulate tick cell biological processes are not known, but may also include epigenetic modifications by pathogen effectors^16^.

In this study, the role of tick oxidative stress during *A*. *phagocytophilum* infection was characterized through the function of different pathways involved in ROS production. The results showed that tick cells increase mt ROS production to limit *A*. *phagocytophilum* infection, while pathogen inhibits alternative ROS production pathways and apoptosis to preserve cell fitness and facilitate infection. The results supported that *A*. *phagocytophilum* uses common mechanisms for infection of ticks and vertebrate hosts, but with differences that could be associated with host-specific cell tropism during pathogen life cycle.

## Results

### A. *phagocytophilum* infection affects tick mt ROS response in a global and tissue-specific manner

**Figure 1:**
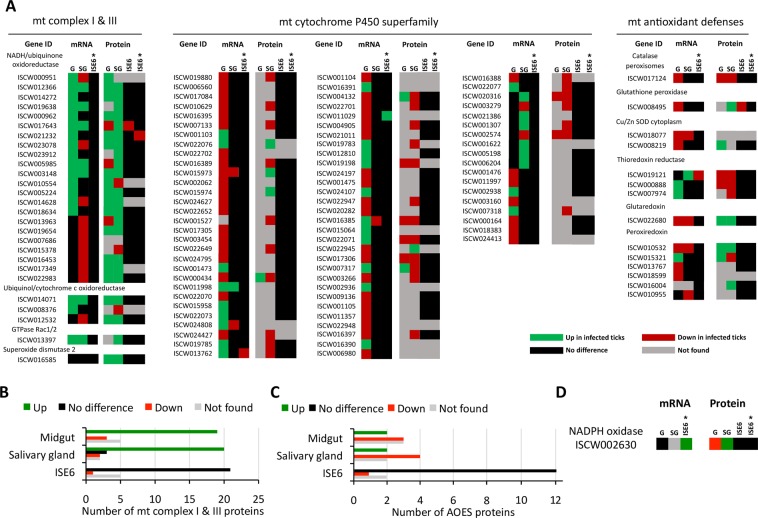
Profile of *I*. *scapularis* mt ROS metabolic enzymes mRNA and protein levels and tick mt oxidative stress in response to *A*. *phagocytophilum* infection. Transcriptomics and proteomics data were obtained from previously published datasets in analyses conducted in *I*. *scapularis* female midguts (G) and female salivary glands (SG) in response to *A*. *phagocytophilum* infection^33,36^. ISE6 cells proteomics data was generated *de novo* after 2 dpi (ISE6) and 7 dpi (ISE6*). Up and Down refer to mRNA/protein levels in infected samples when compared to uninfected controls (P < 0.05). (**A**) Comparison of mt ROS metabolic enzymes mRNA and protein levels. (**B**) mt complex I and III enzymes and (**C**) antioxidant enzymes (AOES) protein levels in tick midgut, salivary gland and ISE6 cells were considered to provide an indicator of the effect of *A*. *phagocytophilum* infection on tick mt ROS metabolic pathways. (**D**) mRNA and protein levels for the putative tick NADPH oxidase (ISCW002630, B7PDQ2).

The putative mt ROS metabolic pathways and the effect of *A*. *phagocytophilum* infection in *I*. *scapularis* adult fed female midguts and salivary glands and ISE6 tick cells which constitute a model for hemocytes^33^ were characterized (Fig. 1A–D). These tissues were selected because of their role during pathogen life cycle in ticks, midgut-pathogen entry, ISE6 cells/hemocytes-pathogen transporter and salivary glands-pathogen exit^34,35^. The results evidenced the effect of infection on the levels of multiple mRNAs and proteins involved in both mt ROS production and antioxidant defenses (Fig. 1A–D). As in previous reports^16–18,33,36^, the transcriptomics and proteomics results showed differences among ISE6 cells, tick midguts and salivary glands in response to *A*. *phagocytophilum* infection. The proteomics results showed that various proteins were not identified in one or several samples (Fig. 1A), probably due to low protein levels in these cells or tissues. However, the levels some proteins were found to change in response to infection (Fig. 1A). Considering the protein levels to provide an indicator of the effect of *A*. *phagocytophilum* infection on tick mt ROS metabolic pathways, the results showed an increase in complex I and III enzymes (Fig. 1B) and a decrease in antioxidant enzymes (AOES) (Fig. 1C) in tick midgut and salivary glands. In ISE6 cells, the results show that the level of most proteins did not change in response to *A*. *phagocytophilum* infection after 2 and 7 days post-infection (2 and 7 dpi, Fig. 1B,C). After 2 dpi, the enzymes NADH ubiquinone oxidoreductase (ISCW017643) and glutathione peroxidase (ISCW008495) were underrepresented in infected ISE6 cells (Fig. 1A). However, after 7 dpi, the levels of these two proteins were not different to that of uninfected cells, but then the enzyme NADH-cytochrome B5 reductase (ISCW021232) was underrepresented in infected cells (Fig. 1A). The absence of correlation between the changes in mRNA and protein levels is a well-known phenomenon described in many other systems^37–44^. In the context of *A*. *phagocytophilum* infection, this mismatch could be due, among other reasons, to delay between transcription and translation and/or the role for post-transcriptional and post-translational modifications in tick cells^16–18,36^.

**Figure 2:**
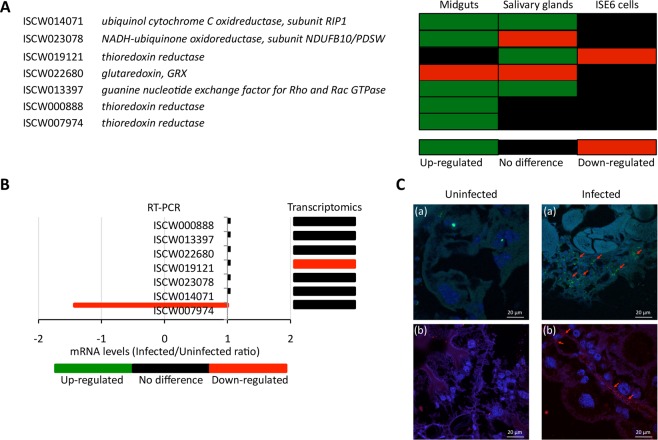
Validation of transcriptomics data by RT-PCR in *A*. *phagocytophilum*-infected and uninfected *I*. *scapularis* ISE6 cells. (**A**) Infected/Uninfected ratio differential expression (P < 0.05) for selected genes involved in mt ROS pathways in *I*. *scapularis* female midguts, salivary glands and ISE6 cells 7 dpi. (**B**) The expression of selected genes was characterized by real-time RT-PCR using total RNA extracted from infected and uninfected ISE6 cells 7dpi. The mRNA levels were normalized against tick 16S rRNA and *cyclophilin* using the genNorm method. Normalized Ct values were compared between infected and uninfected samples by Student’s t-test with unequal variance (P < 0.05; N = 4 biological replicates). The infected to uninfected ratio was calculated by dividing the mean normalized Ct values between infected and uninfected ISE6 cells and compared to transcriptomics data. (**C**) Immunofluorescence assays in *A*. *phagocytophilum*-infected and uninfected *I*. *scapularis* tick midgut digestive cells showed that ubiquinol/cytochrome c oxidoreductase levels (red arrows) were higher in infected ticks. Adult female tick slides showing midgut cells were incubated with anti-ubiquinol-cytochrome c reductase core protein I antibodies (ab96333) and developed with either (a) goat anti-rabbit IgG conjugated with FITC (green) or (b) goat anti-rabbit IgG conjugated with PE (red). Sections of uninfected ticks obtained under similar conditions as infected ticks were used as controls. Nuclei were stained with DAPI (blue). The slides were examined using a Zeiss LSM 800 laser scanning confocal microscope with x40 oil immersion objectives. Bars, 20 µm.

The datasets used in this analysis on the tick transcriptomics (i.e. midguts, salivary glands and ISE6 cells) and proteomics (i.e. midguts and salivary glands) response to *A*. *phagocytophilum* infection have been validated in several studies^16–18,33,36^. Nevertheless, the mRNA levels of selected tick genes differentially expressed in response to *A*. *phagocytophilum* infection (Fig. 2A) were analyzed by quantitative RT-PCR in uninfected and *A*. *phagocytophilum*-infected ISE6 cells, providing additional validating support for 71% of the selected genes (Fig. 2B). At the protein level, the immunofluorescence assay (IFA) in midgut cells from *A*. *phagocytophilum*-infected and uninfected adult *I*. *scapularis* fed females validated proteomics results (Fig. 1A) by showing that ubiquinol/cytochrome c oxidoreductase levels increased in response to infection (Fig. 2C). For analysis, tick midguts were dissected from adult female ticks removed from the sheep 7 days after experimental infestation and washed in PBS to remove hemolymphs-related cells^36^.

### Oxidative stress increases with *A*. *phagocytophilum* infection of ISE6 tick cells to limit pathogen infection

**Figure 3:**
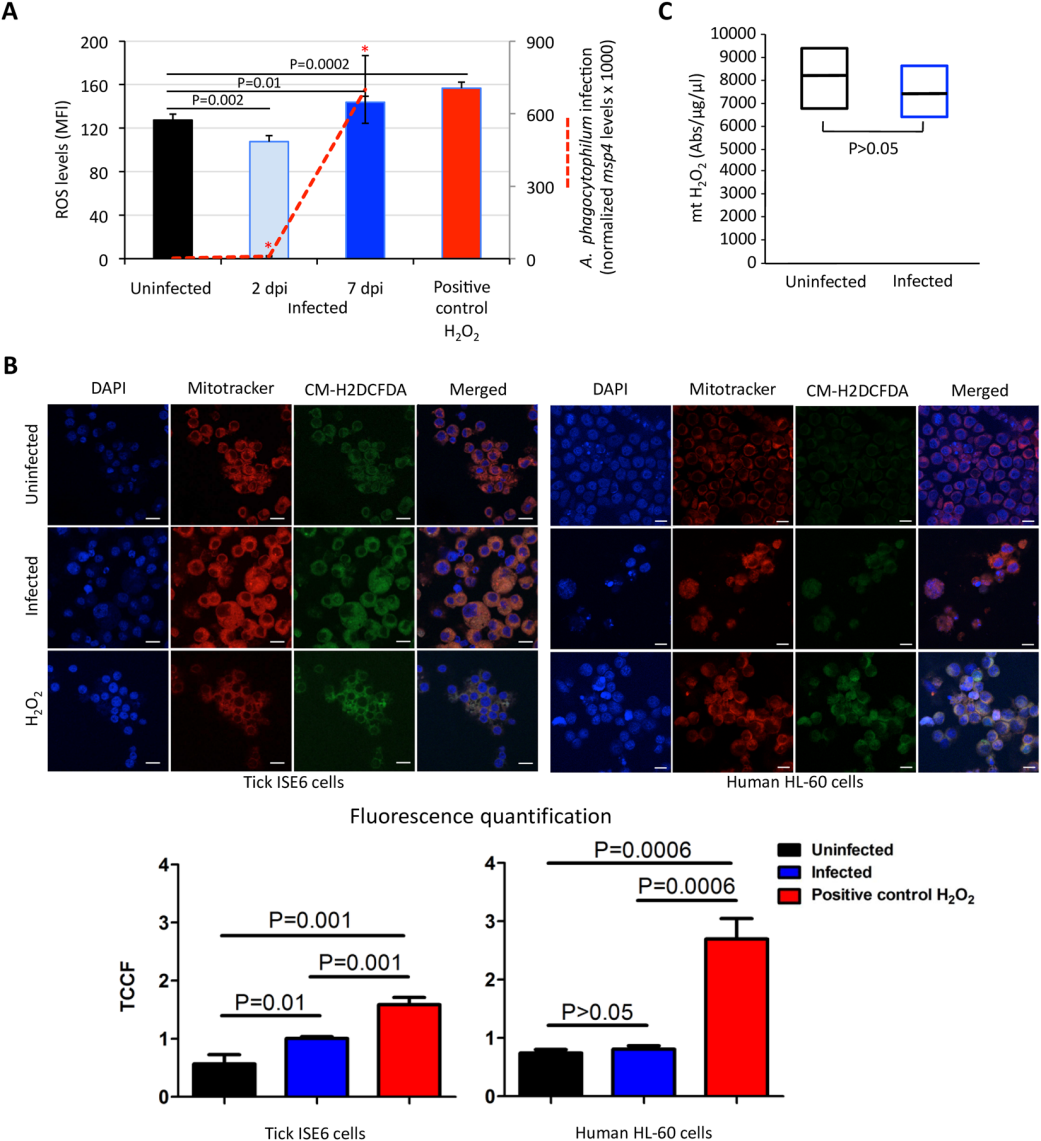
Oxidative stress increases in *A*. *phagocytophilum*-infected tick cells. (**A**) The effect of *A*. *phagocytophilum* infection on ROS generation in ISE6 tick cells was determined using CM-H_2_DCFDA that offers derivatives of reduced fluorescein as cell-permeant indicators for ROS. The ISE6 tick cells were infected with cell-free *A*. *phagocytophilum* (NY18 isolate) and analyzed at 2 and 7 days post-infection (dpi). As a positive control, uninfected cells were treated with 50 µM hydrogen peroxide (H_2_O_2_) for 30 min at 31 °C before ROS detection. The level of ROS in the viable cells was determined as the geometric median fluorescence intensity (MFI). The *A*. *phagocytophilum* infection (red line) was determined by *msp4* PCR normalizing against tick 16S rRNA. The MFI and normalized Ct values (Ave + S.D) were compared between groups by Student’s t-test with unequal variance (P < 0.05; N = 4 biological replicates). (**B**) Fluorescence microscopy detection of mitochondria (mitotracker, red) and intracellular ROS (CM-H2DCFDA, green) in human and tick cells uninfected, *A*. *phagocytophilum*-infected at 7 dpi and treated with 50 µM H_2_O_2_ as a positive control. Nuclei were stained with DAPI (blue). Bars, 10 µm. Using ImageJ, an outline was drawn around each cell and area, mean fluorescence and integrated density were measured, along with several adjacent background readings. The total corrected cellular fluorescence (TCCF) = integrated density – (area of selected cell × mean fluorescence of background readings), was calculated. (**C**) The mt H_2_O_2_ generation was measured using a spectrofluorometer in mitochondria isolated from tick cells uninfected and *A*. *phagocytophilum*-infected at 7 dpi. Data was normalized by protein concentration and compared between groups by Student’s t-test with unequal variance (P < 0.05; N = 3 biological replicates).

To characterize the oxidative stress response to *A*. *phagocytophilum* infection of tick cells, the generation of ROS was determined in infected and uninfected ISE6 cells using CM-H_2_DCFDA. The results showed that while at 2 dpi (10% infected cells), ROS levels were lower in infected than in uninfected cells, the increase in *A*. *phagocytophilum* infected cells at 7 dpi (25% infected cells) resulted in higher ROS levels when compared to uninfected cells (Fig. 3A). These results were supported by fluorescence microscopy detection of mt ROS in cells uninfected and *A*. *phagocytophilum*-infected at 7 dpi showing that mt ROS levels increased in response to infection in ISE6 cells. In contrast to tick cells, the ROS levels did not changed significantly in undifferentiated (Fig. 3B) and differentiated (Fig. S1) HL-60 human cells in response to *A*. *phagocytophilum* infection 7 dpi. The increase of ROS levels in tick cells was also confirmed 7 dpi by fluorescence microscopy using the genetically encoded redox biosensor for H_2_O_2_, roGFP2-Orp1 (Fig. S1). The spectrofluorometric measurement of mt H_2_O_2_ generation in mitochondria isolated from ISE6 tick cells uninfected and *A*. *phagocytophilum*-infected at 7 dpi did not show differences in mt H_2_O_2_ levels in response to infection (Fig. 3C).

**Figure 4:**
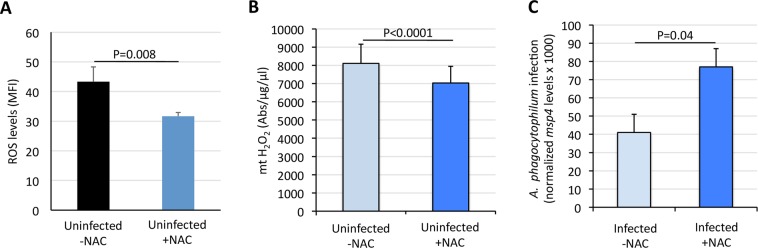
Oxidative stress increases with *A*. *phagocytophilum* infection of ISE6 tick cells to limit pathogen infection. (**A**) The ROS production was determined after 7 days treatment with NAC of uninfected ISE6 tick cells. The level of ROS in the viable cells was determined as the geometric median fluorescence intensity (MFI) using CM-H_2_DCFDA. The MFI values (Ave + S.D) were compared between groups by Student’s t-test with unequal variance (P < 0.05; N = 4 biological replicates). (**B**) The mt H_2_O_2_ generation was measured using a spectrofluorometer in mitochondria isolated from uninfected tick cells untreated and treated with NAC for 7 days. Data was normalized by protein concentration and compared between groups by Student’s t-test with unequal variance (P < 0.05; N = 3 biological replicates). (**C**) The protective effect of ROS in infected tick cells was determined using N-acetyl cysteine (NAC) to inhibit ROS production and evaluate the effect on *A*. *phagocytophilum* infection at 7 dpi. The *A*. *phagocytophilum* infection was determined by *msp4* PCR normalizing against tick 16S rRNA. Normalized Ct values (Ave + S.D) were compared between groups by Student’s t-test with unequal variance (P < 0.05; N = 4 biological replicates).

The protective effect of ROS in infected tick cells was characterized using N-acetyl cysteine (NAC) to inhibit ROS production. NAC, the most powerful cellular antioxidant, is an amino acid that acts in cells as an antioxidant agent as it reacts with ROS such as H_2_O_2_ and OH-^45–47^. ROS levels, measured using CM-H_2_DCFDA, decreased by 27% after treatment of uninfected ISE6 tick cells with NAC for 7 days (Fig. 4A). Similarly, ROS levels, measured using roGFP2-Orp1, decreased significantly after treatment of uninfected ISE6 tick cells with NAC for 2 days (Fig. S2). The proportion of uninfected live ISE6 cells did not changed significantly 7 days after NAC treatment (Fig. S3). However, the proportion of dead cells increased and the proportion of necrotic and apoptotic cells decreased 7 days after NAC treatment (Fig. S3). These results suggest that NAC decreases ROS levels which in turn reduces apoptosis and necrosis without affecting tick cell viability. The mt H_2_O_2_ levels decreased in uninfected tick cells treated for 7 days with NAC when compared to untreated cells (Fig. 4B). In response to decrease in ROS levels, the results showed an increase in *A*. *phagocytophilum* infection in cells inhibited for ROS production at 7 dpi (Fig. 4C).

### The mt ROS production pathways function to limit *A*. *phagocytophilum* infection and multiplication in tick cells

To functionally characterize the role of tick mt ROS production pathways during *A*. *phagocytophilum* infection, pharmacological studies were conducted in ISE6 tick cells using a ROS inducer and inhibitor.

**Figure 5:**
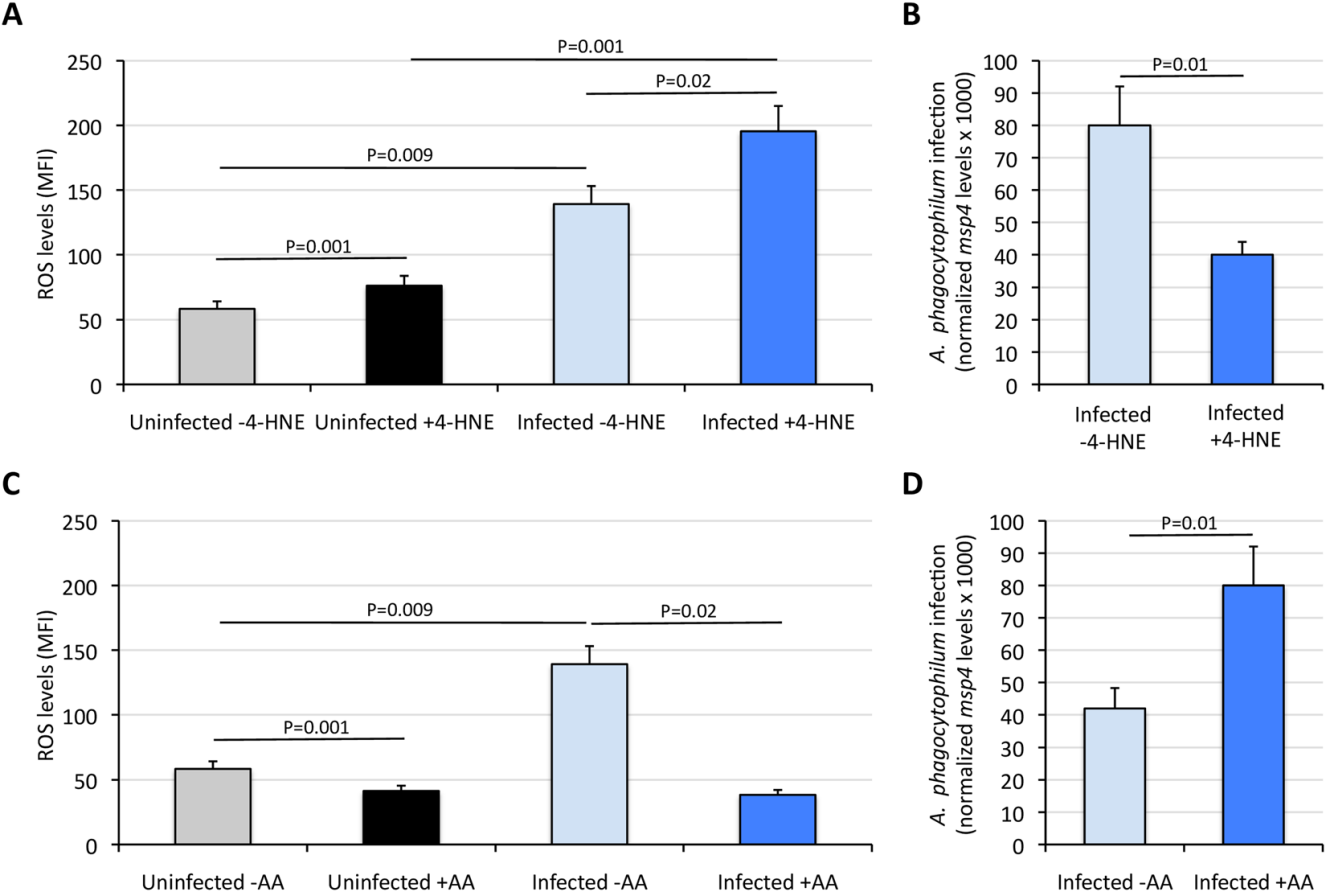
Effect of Antymicin A and 4-Hydroxy-2-nonenal on *A*. *phagocytophilum* infection and ROS levels in ISE6 tick cells. ISE6 tick cells were uninfected or infected with cell-free *A*. *phagocytophilum* (NY18 isolate), treated with AA, 4-HNE or left untreated and analyzed at 7 days post-infection (dpi). (**A**,**C**) The effect of *A*. *phagocytophilum* infection on ROS generation in ISE6 tick cells was determined using CM-H_2_DCFDA. The level of ROS in the cells was determined as the median fluorescence intensity (MFI). (**B**,**D**) The *A*. *phagocytophilum* infection levels were determined by *msp4* PCR normalizing against tick 16 *S rDNA*. Four replicates were done for each assay and MFI and normalized Ct values (Ave + S.D) were compared between groups by Student’s t-test with unequal variance (P < 0.05). Abbreviations: AA, Antymicin A; 4-HNE, 4-Hydroxy-2-nonenal.

The 4-Hydroxy-2-nonenal (4-HNE) is reported to have physiological and cytotoxic effects^48,49^. At micromolar concentrations (<2 µM), 4-HNE has anti-ROS and anti-apoptotic effects, and up-regulates the expression of transcription factors such as NF-κB, which controls the expression of genes involved in cell multiplication and differentiation^48–50^. However, at greater concentration (>10 µM) 4-HNE inhibits NF-κB and induces production of ROS and mt dysfunction^48,49^. Additionally, transactivation of gene expression by NF-κB is dependent on thioredoxin reductase activity^51^, which is inhibited by 4-HNE^52^. In agreement with previous results^48,49^, treatment of ISE6 tick cells with 100 µM 4-HNE for 7 days resulted in an increase in ROS levels, measured by CM-H_2_DCFDA, in both uninfected and infected cells (Fig. 5A). In correspondence with the increase in ROS, *A*. *phagocytophilum* infection levels decreased with 4-HNE treatment at 7 dpi (Fig. 5B). Antimycin A (AA) inhibits mt ubiquinol/cytochrome c oxidoreductase and reduces the production of ROS^53^. Treatment of ISE6 tick cells with 2 μM AA reduced ROS production, measured by CM-H_2_DCFDA, in both uninfected and infected tick cells (Fig. 5C). In response to reduction in ROS levels after AA treatment, the *A*. *phagocytophilum* infection levels increased in AA-treated cells when compared to untreated controls (Fig. 5D).

**Figure 6:**
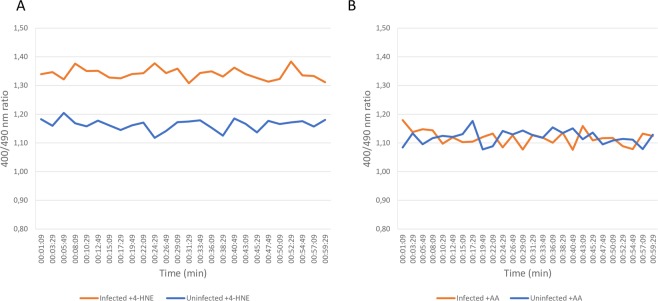
Biosensor redox state. The roGFP2-Orp1 probe redox state was monitored for 60 min in *I*. *scapularis* ISE6 tick cells treated for 2 days with either 100 μM 4-Hydroxy-2-nonenal (**A**) or 2 μM Antimycin A (**B**).

ROS production in infected and uninfected ISE6 tick cells treated and untreated with 4-HNE and AA was also measured 2 dpi using roGFP2-Orp1 (Fig. S4). The results show that *A*. *phagocytophilum* infection increases ROS levels in both 4-HNE-treated and untreated cells (Fig. S4). Higher ROS production in 4-HNE-treated cells was also associated with lower *A*. *phagocytophilum* infection levels (Fig. S4). However, in contrast to 7 dpi, no change was observed in ROS levels after treatment with AA in *A*. *phagocytophilum*-infected and uninfected ISE6 cells and, in consequence, *A*. *phagocytophilum* infection levels were not affected (Fig. S4). Biosensor redox state was also followed for 60 mins and the results showed that ROS levels induced by *A*. *phagocytophilum* infection in AA- and 4-HNE-treated ISE6 cells remains relatively constant over this time (Fig. 6).

**Figure 7:**
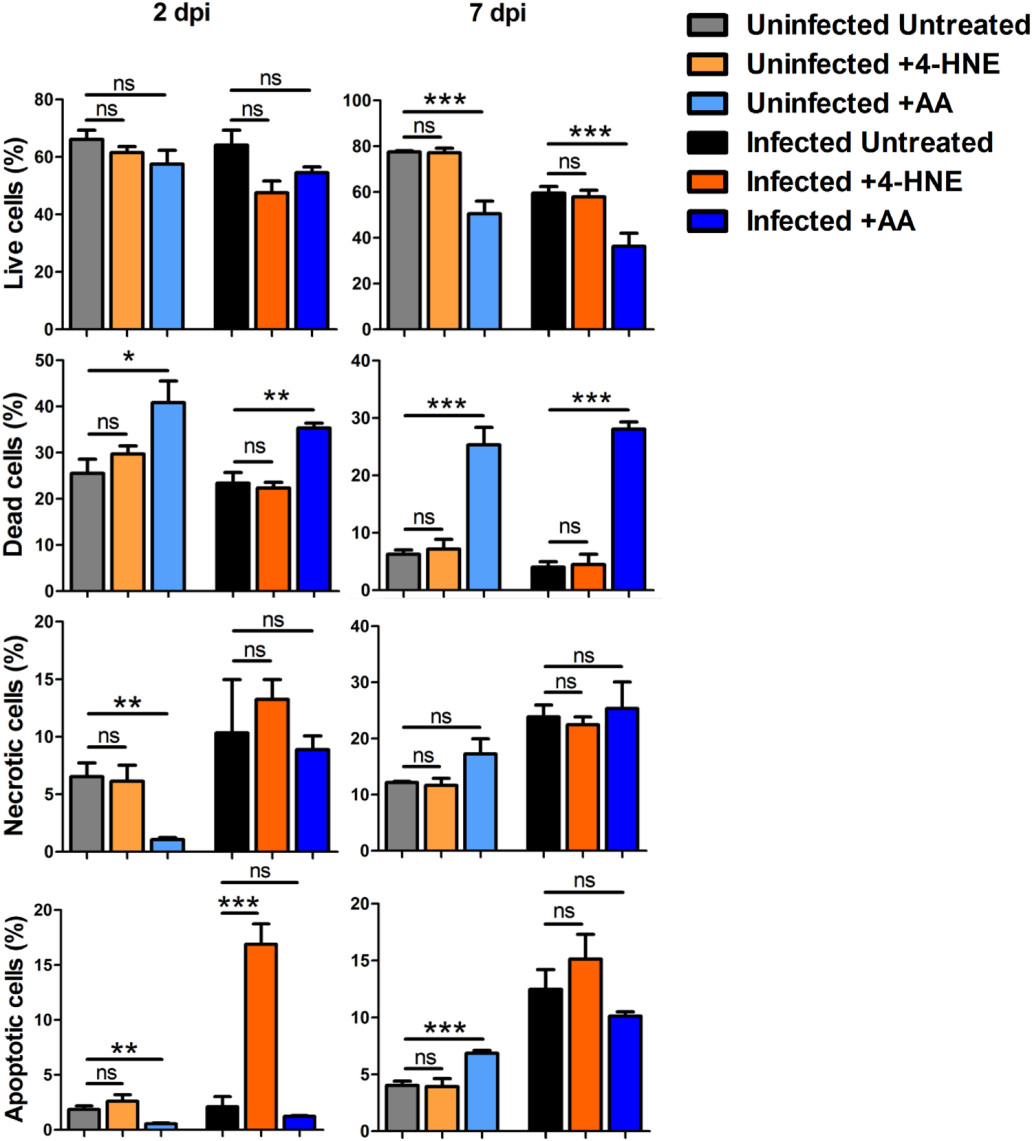
Effect of treatment with Antymicin A and 4-Hydroxy-2-nonenal on the viability of ISE6 tick cells. The percentage of apoptotic, dead/late apoptotic, necrotic and viable cells was determined in AA- (2 µ M) and 4-HNE (100 µM)-treated and untreated infected and uninfected cells by flow cytometry after Annexin V-FITC and PI labeling, and the average of 4 replicated represented. Abbreviations: AA, Antymicin A; 4-HNE, 4-Hydroxy-2-nonenal.

To exclude the possibility that changes in ROS or bacterial levels observed after 4-HNE and AA treatment may be influenced by cellular mortality processes, the proportion of live, dead, necrotic and apoptotic cells was measured in treated, untreated, infected and uninfected tick cells after 2 dpi and 7 dpi (Fig. 7). The treatment of tick cells with AA reduced cell viability at 7 dpi in both uninfected and *A*. *phagocytophilum*-infected cells (P < 0.05), mainly due to increase in dead but not apoptotic or necrotic cells (Fig. 7). In contrast to AA, the treatment with 4-HNE did not affect tick cell viability at 2 dpi or 7 dpi. These results suggest that 4-HNE is not toxic for tick cells, while AA exerts toxic effects when long-term treatments (i.e. 7 dpi) are used in these cells. In addition, the fact that 4-HNE increases (Fig. 5A) and AA (Fig. 5C) and NAC (Fig. 4A,B) decrease ROS levels and 4-HNE increases (Fig. 7) and AA does not affect (Fig. 7) and NAC decreases (Fig. S3) apoptosis, respectively, suggests that the effect of these drugs on bacterial levels (Figs 4C and 5B,D) is mediated by the regulation of apoptosis. In consequence, an increase in ROS levels increases apoptosis which in turn impedes *A*. *phagocytophilum* infection, while a decrease in ROS levels decreases or does not affect apoptosis which in turn facilitates *A*. *phagocytophilum* infection.

### *A*. *phagocytophilum* affects ROS production by similar mechanisms in tick cells and vertebrate host neutrophils

Recent results suggested that *A*. *phagocythophilum* uses similar mechanisms for infection and multiplication in tick and vertebrate host cells^14^. In vertebrate neutrophils, the infection with *A*. *phagocytophilum* does not induce the production of ROS to facilitate pathogen infection and multiplication^19,20^. How pathogen infection prevents mt ROS production in neutrophils has not been fully characterized. However, the mechanism used by *A*. *phagocytophilum* to inhibit intracellular ROS production in neutrophils and HL-60 cells appears to be caused by the down-regulation of NADPH oxidase components^19–23^, mediated at least in part by the pathogen effector AnkA-dependent effect on gene expression^23^. However, the effect of pathogen infection on the NADPH oxidase-mediated ROS production has not been characterized in ticks.

To address this question, the mRNA and protein levels for the putative tick NADPH oxidase (ISCW002630, B7PDQ2) with 41% identity (*E*-value 8e-171) to human NADPH oxidase 5 NOX5 protein (AAI25099) were characterized in uninfected and *A*. *phagocytophilum* infected *I*. *scapularis* midguts and salivary glands using the available transcriptomics and proteomics data. Tick NADPH oxidase mRNA and protein levels in ISE6 cells were determined using available (mRNA, 7 dpi) and *de novo* proteomics analysis after 2 and 7 dpi (Fig. 1D). The results showed that in tick midgut, but not in salivary glands, protein levels decreased in response to infection, with a possible impact on reduced ROS production and apoptosis, which may favor pathogen infection (Fig. 1D). In contrast, tick NADPH oxidase protein levels in ISE6 cells did not changed at 2 or 7 dpi (Fig. 1D). At the transcriptional level it was not possible to show gene down-regulation as the mechanism responsible for reduction in NADPH oxidase protein levels in guts (Fig. 1D), which may be due to different factors affecting the correlation between mRNA and protein levels. Furthermore, the analysis of bacterial AnkA/Ank protein levels in *A*. *phagocytophilum*-infected *I*. *scapularis* midgut, salivary glands and ISE6 cells using previously published results^33,36^ showed the presence of these proteins identified with 246, 573 and 396 peptide spectrum matches (PSM), respectively (Table S1). Additionally, in a separate experiment with infected ISE6 tick cells^54^, Ank proteins were also identified with a similar number of PSM (N = 397; Table S1). Future experiments should address the possible involvement of *A*. *phagocytophilum* AnkA/Ank proteins in the regulation of the mt ROS production.

## Discussion

The transcriptomics and proteomics analysis of mt ROS response of *I*. *scapularis* adult fed female midguts and salivary glands and ISE6 cells in response to *A*. *phagocytophilum* infection supported the presence of tissue-specific differences in the tick cell response to infection^16–18,33,35,36,55^, and suggested a global effect of *A*. *phagocytophilum* infection on tick redox metabolic pathways. In particular, the analysis of tick mt ROS metabolic pathways protein levels in response to *A*. *phagocytophilum* infection showed an increase in complex I and III enzymes involved in ROS production and a decrease in AOES. The differences in the expression/representation between different cytochrome P450 superfamily genes/proteins probably reflected the still uncharacterized role that these enzymes play in tick ROS production and antioxidant defenses^1,9,10,25^.

The results of pharmacological studies with ROS inductors and inhibitors supported that mt ROS production pathways function to limit *A*. *phagocytophilum* infection and multiplication in tick cells. The use of ROS inductors such as 4-HNE together with the finding that certain thioredoxin reductases were down-regulated at the mRNA and/or protein levels in tick midguts, salivary glands and ISE6 cells suggested that tick cells reduce mt antioxidant defenses mediated by thioredoxin reductase and other enzymes such as catalase peroxisomes, Cu/Zn SOD and glutaredoxin to favor ROS accumulation and limit *A*. *phagocytophilum* infection. The results with the ROS inhibitor AA and the increase of mRNA and protein levels of complex III enzymes in tick midguts and salivary glands suggests that mt ROS production by ubiquinol/cytochrome c oxidoreductases in part of the tick response to limit *A*. *phagocytophilum* infection in tick cells.

**Figure 8:**
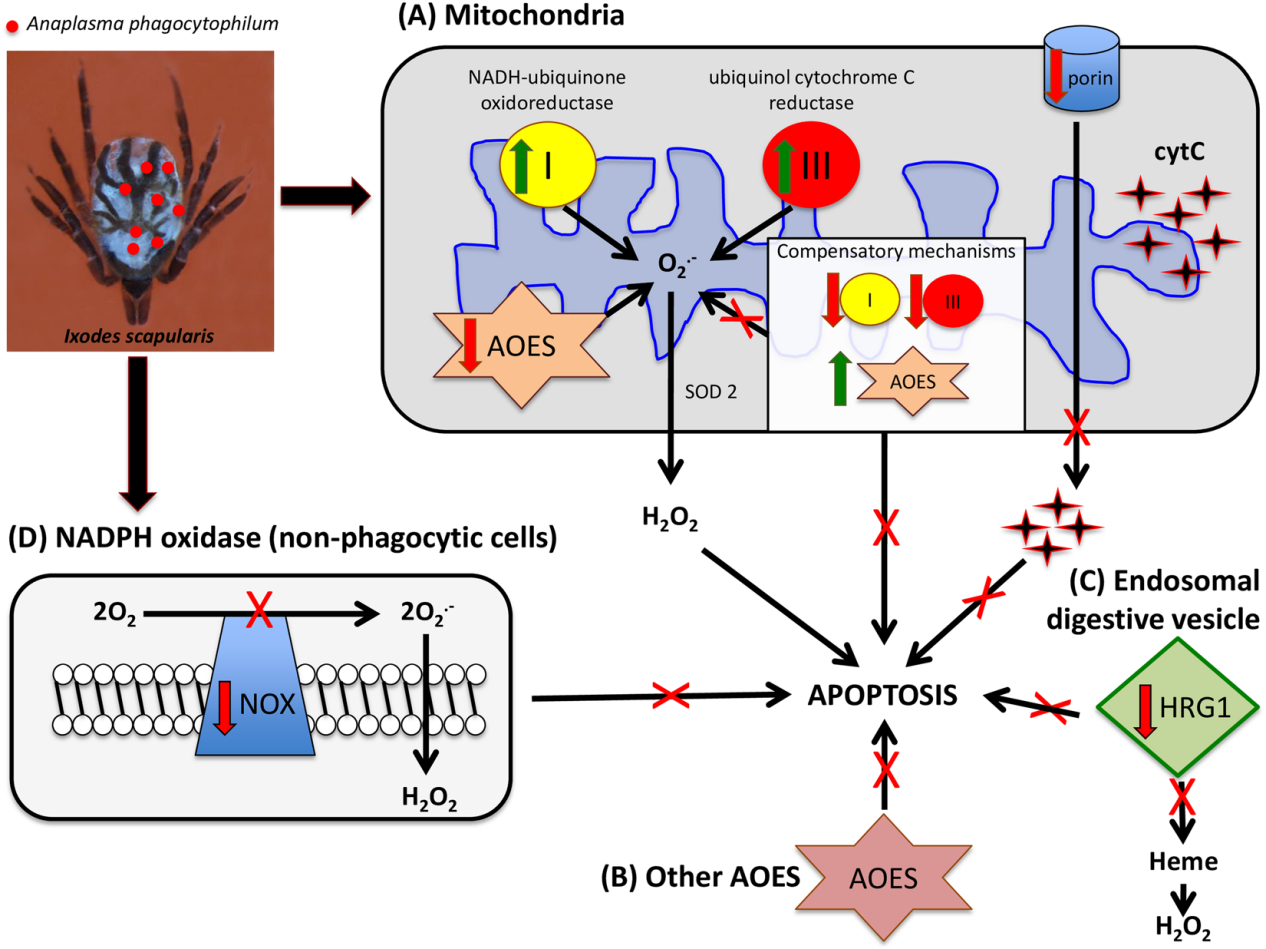
Model of the function of tick mt ROS production pathways during *A*. *phagocytophilum* infection. Taking together the results of the tick transcriptome and proteome in response to *A*. *phagocytophilum* infection and the functional studies conducted here, we proposed a model for the role of ROS production during pathogen infection and multiplication in ticks. (**A**) The increase in protein levels of mt enzymes of the NADH-ubiquinone oxidoreductase (complex I) and ubiquinol cytochrome C reductase (complex III) together with the down-regulation at the mRNA and/or protein levels of AOES in response to *A*. *phagocytophilum* infection probably resulted in the generation of ROS and induced apoptosis as protective mechanisms against infection. However, the decrease in the levels of certain mt complex I and III enzymes and/or the increase in some AOES probably reflected a compensatory mechanism to reduce ROS production/accumulation and preserve cell function and tick fitness while limiting pathogen infection. ROS induction of the intrinsic apoptosis pathways was inhibited by *A*. *phagocytophilum* through Porin down-regulation, favoring bacterial infection (**B**) Other non-mt AOES may also function to control redox homeostasis, antioxidant defense and ROS detoxification. (**C**) The effect of *A*. *phagocytophilum* infection on the under-representation of tick HRG1 suggested a mechanism activated by the pathogen in the endosomal digestive vesicle to reduce the antimicrobial oxidative burden caused by ROS generated after heme release. (**D**) In tick midgut and ISE6 cells but not in salivary glands, NADPH oxidase protein levels decreased in response to infection, with a possible impact on reduced ROS production and apoptosis, which may favor pathogen infection.

Taking together the results of the variations in tick transcriptome and proteome in response to *A*. *phagocytophilum* infection and the functional studies conducted here, we proposed a model for the role of ROS production during pathogen infection and multiplication in ticks (Fig. 8A–D). The inhibition of tick cell apoptosis is a physiologically relevant mechanism used by *A*. *phagocytophilum* to facilitate infection and multiplication in both tick and vertebrate host cells^14,15,56^. The increase in protein levels of mt enzymes of the NADH-ubiquinone oxidoreductase (complex I) and ubiquinol cytochrome C reductase (complex III) in response to *A*. *phagocytophilum* infection (Fig. 1A,B), probably resulted in the generation of ROS and induced apoptosis as protective mechanisms against infection (Fig. 8A). At the same time, the down-regulation at the mRNA and/or protein levels of AOES (Fig. 1A,C) suggested that tick cells reduce mt antioxidant defenses to favor ROS accumulation and limit *A*. *phagocytophilum* infection (Fig. 8A). However, the decrease in the levels of certain mt complex I and III enzymes and/or the increase in some AOES probably reflected a compensatory mechanism to reduce ROS production/accumulation and preserve cell function and tick fitness while limiting pathogen infection (Fig. 8A). Furthermore, other non-mt AOES such as those up-regulated in tick midgut in response to blood feeding may also function to control redox homeostasis, antioxidant defense and ROS detoxification^25^ (Fig. 8B). Finally, the effect of *A*. *phagocytophilum* infection on the under-representation of tick HRG1^31^ suggested a mechanism activated by the pathogen in the endosomal digestive vesicle to reduce heme release into the cytoplasm of midgut cells to facilitate infection through reduction of the antimicrobial oxidative burden caused by ROS generated after heme release (Fig. 8C).

ROS induce cell death by promoting the intrinsic apoptosis pathway^2^, but *A*. *phagocytophilum* inhibits the intrinsic apoptosis pathway in tick salivary glands and ISE6 cells through Porin down-regulation that resulted in the inhibition of Cytochrome c release as the anti-apoptotic mechanism to facilitate bacterial infection^35,36^ (Fig. 8A). These results evidenced how the tick cell response through ROS induction of intrinsic apoptosis pathway to limit pathogen infection is counteracted by *A*. *phagocytophilum* though a different mechanism to inhibit apoptosis and facilitate infection and multiplication in ticks. The production of ROS in response to infection may results in cell dead, which will also interrupt pathogen life cycle and evidences a conflict between ticks and *A*. *phagocytophilum*^32^. However, ticks and *A*. *phagocytophilum* may benefit from pathogen inhibition of ROS production and apoptosis to preserve cell viability and facilitate infection, providing a ground for cooperation between both organisms^32^. The characterization of tick cell viability in response to different compounds (4-HNE and AA) and *A*. *phagocytophilum* infection suggested that the production of ROS occurs at intermediate levels for cell adaptation to stress and survival. The balance between tick mt oxidative stress in response to infection and the inhibition of ROS production by *A*. *phagocytophilum* using alternative pathways such as reduction in NADPH oxidase levels (Fig. 8D) and the selective manipulation of host proteins to reduce heme release and the antimicrobial oxidative response^31^ facilitates survival of both ticks and pathogens. In summary, modeling the function of tick mt ROS production pathways suggested the activation of oxidative stress and apoptosis to limit *A*. *phagocytophilum* infection while compensatory mechanisms are activated to limit ROS production and accumulation to preserve cell function and tick fitness.

These results supported that *A*. *phagocytophilum* uses common mechanisms for infection of tick vectors and vertebrate hosts^14^. The inhibition of NADPH oxidase-mediated ROS production by pathogen infection appears to occur in both neutrophils and tick cells. However, the inhibition of ROS possibly also occurs through different pathways and at different levels in neutrophils and tick cells. A question remains whether *A*. *phagocytophilum*-derived Ank or other effectors mediate the reduction in the levels of NADPH oxidase and/or other ROS pathway proteins in ticks.

Differences in ROS response to *A*. *phagocytophilum* infection between human and tick cells may reflect host-specific cell tropism that evolved during pathogen life cycle. In ticks, *A*. *phagocytophilum* infects several cell types, while in vertebrate hosts the pathogen infects only neutrophils and therefore a higher molecular tolerance should be expected^13,33,34,57^. These results may be relevant for designing effective intervention to reduce pathogen infection in vertebrate hosts and tick vectors.

## Materials and Methods

### Ethics statement

*Ixodes scapularis* ticks were obtained from the laboratory colony maintained at the Oklahoma State University Tick Rearing Facility. Larvae and nymphs were fed on rabbits and adults were fed on sheep. Off-host ticks were maintained in a 12 hours light: 12 hours dark photoperiod at 22–25 °C and 95% relative humidity. Adult female *I*. *scapularis* were infected with *A*. *phagocytophilum* by feeding on a sheep inoculated intravenously with approximately 1 × 10^7^*A*. *phagocytophilum* (NY18 isolate)-infected HL-60 cells (90–100% infected cells) as previously described^36^. All experiments were performed in accordance with guidelines from the Guide for the Care and Use of Laboratory Animals of the NIH. Animals were housed and experiments conducted with the approval and supervision of the OSU Institutional Animal Care and Use Committee (Animal Care and Use Protocol, ACUP No. VM1026). Every effort was made to reduce distress in animals.

### *I*. *scapularis* ISE6 tick cells and human HL-60 undifferentiated promyelocytic cells

The *I*. *scapularis* embryo-derived tick cell line ISE6 (provided by Professor Ulrike Munderloh) was cultured in L-15B300 medium as previously described^58,59^ and kept at 31 °C. The human promyelocytic HL-60 (ATCC CCL-240) cell line was purchased from American Type Culture Collection (Manassas, VA) and kept at 37 °C in RPMI medium (Gibco, Gaithersburg, MD, USA) supplemented with 10% FBS (FBS, Sigma, MO, USA) as described before^57^. Tick cells were inoculated with *A*. *phagocytophilum* (human NY18 isolate)-infected HL-60 cells that have been cultured in our laboratory for more than 10 years as described previously^33,57,59^. Tick cells collected at 2 and 7 dpi contained 10% and 25% infected cells, respectively. The percentage of cells infected with *A*. *phagocytophilum* was calculated by examining at least 200 cells using a 100x oil immersion objective. Promyelocytic leukemic HL-60 human cells were maintained in RPMI 1640 medium and infected with *A*. *phagocytophilum* (NY18 isolate) as previously described^57^.

### *de novo* proteomics analysis of ISE6 tick cells

Approximately 10^7^ uninfected and *A*. *phagocytophilum* infected ISE6 tick cells at 2 and 7 dpi were washed with phosphate buffered saline (PBS) and resuspended in 500 µl of RIPA Lysis and Extraction Buffer (Thermo Fisher Scientific, Waltham, MA, USA). Samples were sonicated for 1 min in an ultrasonic cooled bath, followed by vortexing for 10 s. After three cycles of sonication–vortex, total cell extracts were centrifuged at 200 × *g* for 5 min to remove cell debris. The supernatants were collected, and protein concentration was determined using the BCA Protein Assay (Life Technologies, Carlsbad, CA) with BSA as standard. Two biological replicates for each condition were prepared.

The protein extracts (50 µg) from uninfected and infected tick cells were *on gel* concentrated and digested with trypsin as described previously^33^. The desalted protein digests were dried, resuspended in 0.1% formic acid and analyzed by reverse phase liquid chromatography coupled to mass spectrometry (RP-LC-MS/MS) in an Easy-nLC II system coupled to an ion trap LTQ-Orbitrap-Velos-Pro hybrid mass spectrometer (Thermo Fisher Scientific, Waltham, MA, USA). The peptides were on line concentrated using a 0.1 mm × 20 mm C18 RP precolumn (Thermo Fisher Scientific, Waltham, MA, USA), and then separated in a 0.075 mm × 250 mm C18 RP column (Thermo Fisher Scientific, Waltham, MA, USA) operating at 0.3 μl/min. Peptides were eluted in a 180-min dual gradient from 5 to 25% solvent B in 135 min followed by gradient from 25 to 40% solvent B over 180 min (Solvent A: 0,1% formic acid in water, solvent B: 0,1% formic acid, 80% acetonitrile in water). ESI ionization was done using a Nano-bore emitter Stainless Steel ID 30 μm (Proxeon) interface. Peptides were detected in survey scans from 400 to 1600 amu (1 μscan) with Orbitrap resolution set at 30.000, followed by twenty data dependent MS/MS scans (Top 20), using an isolation width of 2 u (in mass-to-charge ratio units), normalized collision energy of 35%, and dynamic exclusion applied during 30 s periods.

The MS/MS raw files were searched against a compiled database containing all sequences from *Ixodes scapularis* proteome and *Anaplasma phagocytophilum* taxonomy (20,473 and 21,920 Uniprot entries, respectively, in March 2019) (http://www.uniprot.org) using the SEQUEST algorithm (Proteome Discoverer 1.4, Thermo Fisher Scientific, Waltham, MA, USA). For database searching, parameters were select as follows: trypsin digestion with 2 maximum missed cleavages, tolerances of 800 ppm for precursor ions and 0.8 Da for fragment ions, and variable modifications of Met oxidation and Cys carbamidomethylation. Searches were also performed against a decoy database in an integrated decoy approach. A false discovery rate (FDR) < 0.05 was considered as condition for successful peptide assignments and at least two peptides per protein in at least one of the samples analyzed were the necessary condition for protein identification (Table S2). Two biological replicates were used for each uninfected and infected tick cells and two technical replicated were run per sample. For the quantitative analysis of tick proteins, after discarding Anaplasma proteins in infected cells, the total number of peptide-spectrum matches (PSMs) for each tick protein was normalized against the total number of PSMs in tick cells and compared between uninfected and infected cells by Chi2-test (P < 0.05). The mass spectrometry proteomics data have been deposited at the PeptideAtlas repository (http://www.peptideatlas.org/) with the dataset identifier PASS01351.

### Characterization of the *I*. *scapularis* mRNA and protein levels in response to *A*. *phagocytophilum* infection

The quantitative transcriptomics data for uninfected and *A*. *phagocytophilum*-infected *I*. *scapularis* female midguts and salivary glands and ISE6 cells were obtained from previously published results^33,36^. Quantitative proteomics data for uninfected and *A*. *phagocytophilum*-infected *I*. *scapularis* female midguts and salivary glands was obtained from previously published results^33^. Data deposited at the Dryad repository database, NCBI’s Gene Expression Omnibus database and ProteomeXchange Consortium via the PRIDE partner repository with the dataset identifier PXD002181 and doi: 10.6019/PXD002181. The genes identified in the tick ROS metabolic pathways were searched against the transcriptomics and proteomics data to characterize their mRNA and protein levels in response to *A*. *phagocytophilum* infection.

### Transfection of ISE6 and differentiated HL-60 cells

Before transfection, HL-60 cells were differentiated to a neutrophil phenotype by culturing the cells in medium supplemented with 1.3% DMSO for 5 days^60,61^.

The human isolate of *A*. *phagocytophilum* (strain NY-18) was used to infect human and tick cells as described previously^33,57,59^. On the day of transfection, approximately 1 × 10^5^ cells per well were plated out in 0.5 ml of complete media. For each well to be transfected, 500 ng of pLPCX-roGFP2-Orp1 plasmid DNA^62^ were diluted in 100 µl of Opti-MEM® (Gibco, Waltham, MA, USA) without serum. 1.5 µl per well of Lipofectamine 3000 (Invitrogen) were added to the DNA solution and the mixture was incubated for 25 min at room temperature to allow the formation of complexes. 100 µl of the solution were added directly to each well and the cells were incubated at 37 °C (31 °C for tick cells) in a CO_2_ incubator for 48 hours post-transfection before assaying for transgene expression. pLPCX roGFP2-Orp1 was a gift from Tobias Dick (Addgene plasmid # 64991).

### Microplate-based measurement of roGFP2 fluorescence

roGFP probes display two fluorescence excitation maxima whose relative amplitudes depend on the specific mutations but also depend on the state of oxidation, this can be used to make ratiometric measurements^63^. Transfected tick cells expressing roGFP2 were harvested and washed twice in PBS. Aliquots (100 μL) of cell suspensions were placed in the wells of flat bottom 96-well assay plates (Nunc™ MicroWell™, Thermo Scientific, Waltham, MA, USA). Fluorescence intensity was recorded every 90 seconds for 60 min using excitation filters 400 and 490 nm (10 and 20 nm bandwidth, respectively). A 528 nm (GFP) emission filter (20 nm bandwidth) was accordingly used. Measurements were performed in a multi-detection microplate reader (Cytation 5 Cell Imaging Multi-Mode Reader spectrofluorometer; Biotek, Winooski, VT, USA). The fluorescence excitation ratios (400/490 nm) were used as index for cellular oxidation^64^.

### Imaging of HL-60 and tick cells

Cells were imaged using a Zeiss LSM800 Confocal microscope equipped with an EC Plan 63x objective (oil immersion). Probe fluorescence was excited sequentially at 405 and 488 nm (line by line) and detected at 500–530 nm. Images were saved as 16-bit tif files and processed by ImageJ software. Background was substracted using the rolling ball procedure set to 50 pixels. Pictures were then converted to 32-bit format. A ratio image was created by dividing the 405 nm image by the 488 nm image pixel by pixel and displayed in false colors using the lookup table “Fire” as previously described^65^.

### Pharmacological studies in ISE6 tick cells

ISE6 tick cells uninfected and infected with *A*. *phagocytophilum* were left untreated or treated for 2 and/or 7 days with 2 μM Antimycin A (AA; Sigma-Aldrich, St. Louis, MO, USA) to inhibit Ubiquinol/cytochrome c oxidoreductase^53^, 100 μM 4-Hydroxy-2-nonenal (4-HNE; Invitrogen, Carlsbad, CA, USA) to inhibit Thioredoxin-reductase^52^, and 10 μM N-acetyl cysteine (NAC; Sigma-Aldrich), a free radical scavenger antioxidant agent that reacts with ROS such as H_2_O_2_ and OH-^45–47^. Cells were harvested and used for Annexin V-FITC staining to detect cell viability, as described below, to determine ROS levels and for DNA extraction to determine *A*. *phagocytophilum* DNA levels. All treatments were done in quadruplicate.

### Detection of ROS in *A*. *phagocytophilum*-infected tick and human cells

The effect of *A*. *phagocytophilum* infection on ROS production in ISE6 tick and human cells was determined using CM-H_2_DCFDA (Invitrogen), as it offers derivatives of reduced fluorescein as cell-permeant indicators for ROS. The ISE6 tick cells were seeded (approximately 5 × 10^5^ cells per well; N = 4 biological replicates) in 24-well plates and were infected with cell-free *A*. *phagocytophilum* (NY18 isolate) for 7 days. As a positive control, uninfected tick cells were treated with 50 µM H_2_O_2_ for 30 min at 31 °C before ROS detection^1^. Culture medium was then removed and tick cells were incubated with 5 μM CM-H_2_DCFDA solution in PBS for 40 min at 31 °C. After incubation, cells were washed two times with PBS, collected and then probe oxidation was detected by monitoring changes in fluorescence intensity by flow cytometry. All samples were analyzed on a FAC-Scalibur flow cytometer equipped with CellQuest Pro software (BD Bio-Sciences, Madrid, Spain). The viable cell population was gated according to forward-scatter and side-scatter parameters. The level of ROS in the viable cells was determined as the median fluorescence intensity (MFI). Results were compared between groups by Student’s t-test with unequal variance (P < 0.05; N = 4 biological replicates). For immunofluorescence microscopy, mt from uninfected and infected tick ISE6 and human HL-60 cells were stained with Mitotracker Red (Thermo Scientific, Waltham, MA, USA) and then fixed with 4% paraformaldehyde in PBS for 20 min at room temperature (RT). After washes in PBS, cell smears were prepared using a cytocentrifuge, then mounted in ProLong Antifade with DAPI reagent (Molecular Probes, Eugene, OR, USA) and examined using a Zeiss LSM 800 laser scanning confocal microscope (Carl Zeiss, Oberkochen, Germany) with x60 oil immersion objectives.

### Isolation of mitochondria from tick cells and spectrofluorometric measurement of mt H_2_O_2_ generation

Mitochondria from tick cells were isolated using the mitochondria isolation kit for cultured cells (Thermo Fisher Scientific, Waltham, MA, USA) following a reagent-based method as per manufacturer’s instructions. The mt H_2_O_2_ generation was measured using a spectrofluorometer in mitochondria isolated from tick cells with functioning respiratory chain, active oxidative phosphorilation and catalase, uninfected, *A*. *phagocytophilum*-infected at 7 dpi, and NAC-treated. The H_2_O_2_ released from mitochondria was measured using the Amplex Red hydrogen peroxide/peroxidase assay kit (Invitrogen) following manufacturer’s recommendations. The fluorescence (excitation at 563 nm and emission at 587 nm) was measured at 15 min using a Cytation 5 Cell Imaging Multi-Mode Reader spectrofluorometer (Biotek, Winooski, VT, USA). The total H_2_O_2_ released was corrected for non-specific oxidation of Amplex Red measured in the absence of horseradish peroxidase as previously described^27^, normalized by protein concentration and compared between groups by Student’s t-test with unequal variance (P < 0.05; N = 3 biological replicates).

### Immunofluorescence in adult female ticks

Adult *I*. *scapularis* females were fed on uninfected and *A*. *phagocytophilum* (NY18)-infected sheep as described before^66^. Infected and uninfected female fed ticks were fixed, embedded in paraffin, and sections (4 μm) prepared and mounted on glass slides as previously described^36^. The slides were processed^36^ and then incubated for 14 h at 4 °C with anti-ubiquinol-cytochrome c reductase core protein I antibodies (ab96333; Abcam, Cambridge, UK) diluted 1:100 in 3% BSA/PBS and after 3 washes in PBS, developed for 1 h with either goat-anti-rabbit IgG conjugated with phycoerythrin (PE) (Sigma-Aldrich) 1:50 dilution in 3% BSA/PBS or goat anti-rabbit IgG conjugated with FITC (Sigma-Aldrich) 1:200 dilution in 3% BSA/PBS. Finally, the slides were mounted in ProLong Antifade with DAPI reagent (Molecular Probes) and examined using a Zeiss LSM 800 laser scanning confocal microscope (Carl Zeiss, Oberkochen, Germany) with x40 oil immersion objectives.

### Determination of *A*. *phagocytophilum* DNA levels in ISE6 tick cells

Total DNA was extracted from 200 μl of a tick cell suspension using the RealPure Spin Kit (Durviz, Valencia, Spain) following the manufacturer’s recommendations. *A*. *phagocytophilum* DNA levels were characterized by *msp4* real-time PCR normalizing against tick 16S rRNA as previously described^67^. An F-test of equality of variances (http://www.statskingdom.com/220VarF2.html) was first conducted and normalized Ct values were compared between untreated and treated cells by Student’s t-test with unequal variance (P < 0.05; N = 4 biological replicates).

### Characterization of gene expression by real-time RT-PCR

The expression of selected genes (Table S3) was characterized using total RNA extracted from uninfected and infected ISE6 tick cells using TRIzol (Invitrogen, Carlsbad, CA, USA) following manufacturer´s recommendations. Real-time RT-PCR was performed on RNA samples using gene-specific oligonucleotide primers (Table S3).

Quantitative RT-PCR was performed using a Quantitect SYBR Green RT-PCR Kit and a Rotor Gene Q thermocycler (Qiagen Inc., Valencia, CA, USA) following manufacturer’s recommendations. A dissociation curve was run at the end of the reaction to ensure that only one amplicon was formed and that the amplicon denatured consistently in the same temperature range for every sample^68^. The mRNA values were normalized against tick 16S rRNA and *cyclophilin* using the genNorm method (ddCT)^69^. We used two reference genes to correct for sample to sample variations and improve gene quantification analysis. An F-test of equality of variances (http://www.statskingdom.com/220VarF2.html) was first conducted and normalized Ct values were compared between infected and uninfected tick cells by Student’s t-test with unequal variance (P < 0.05; N = 4 biological replicates).

### Annexin V-FITC staining to detect tick cell viability after experimental infection with *A*. *phagocytophilum*

Approximately 5 × 10^5^ uninfected and *A*. *phagocytophilum*-infected ISE6 tick cells were collected after different treatments. Cell viability (proportion of viable, necrotic, dead/late apoptotic and apoptotic cells) was measured by flow cytometry using the Annexin V-fluorescein isothiocyanate (FITC) apoptosis detection kit (Immunostep, Salamanca, Spain) as previously described^18^. An F-test of equality of variances (http://www.statskingdom.com/220VarF2.html) was first conducted and the percentage of apoptotic, dead/late apoptotoc, necrotic and viable cells was compared between both treated and untreated infected and uninfected cells by Student’s t-test with unequal variance (P < 0.05; N = 4 biological replicates).

## Supplementary information

Supplementary materials

Supplementary Table S1

Supplementary Table S2

Supplementary Table S3

## Data Availability

Quantitative transcriptomics and proteomics data for uninfected and *A*. *phagocytophilum*-infected *I*. *scapularis* female midguts and salivary glands, and ISE6 tick cells are available at the Dryad repository database, NCBI’s Gene Expression Omnibus database and ProteomeXchange Consortium via the PRIDE partner repository with the dataset identifier PXD002181 and doi: 10.6019/PXD002181.
