# Supplementary material for: The redox metabolic pathways function to limit Anaplasma phagocytophilum infection and multiplication while preserving fitness in tick vector cells
Source: Sci Rep. 2019 Sep 13;9:13236. doi: 10.1038/s41598-019-49766-x (PMC6744499; doi:10.1038/s41598-019-49766-x)
Supplement: Supplementary file 1 — Supplementary materials [file 41598_2019_49766_MOESM1_ESM.pdf]

The redox metabolic pathways function to limit *Anaplasma phagocytophilum* infection and multiplication while preserving fitness in tick vector cells

Pilar Alberdi[1,¶], Alejandro Cabezas-Cruz[2,¶,*], Pedro Espinosa Prados[1,¶], Margarita Villar Rayo[1], Sara Artigas-Jerónimo[1], José de la Fuente[1,3*]

[1]SaBio. Instituto de Investigación en Recursos Cinegéticos IREC (CSIC-UCLM-JCCM), 13005 Ciudad Real, Spain; [2]UMR BIPAR, INRA, ANSES, Ecole Nationale Vétérinaire d'Alfort, Université Paris-Est, Maisons-Alfort, 94700, France; [3]Department of Veterinary Pathobiology, Center for Veterinary Health Sciences, Oklahoma State University, Stillwater, OK 74078 USA

[¶]Equal contribution

*** Correspondence:** Alejandro Cabezas-Cruz, cabezasalejandrocruz@gmail.com; José de la Fuente, jose_delafuente@yahoo.com

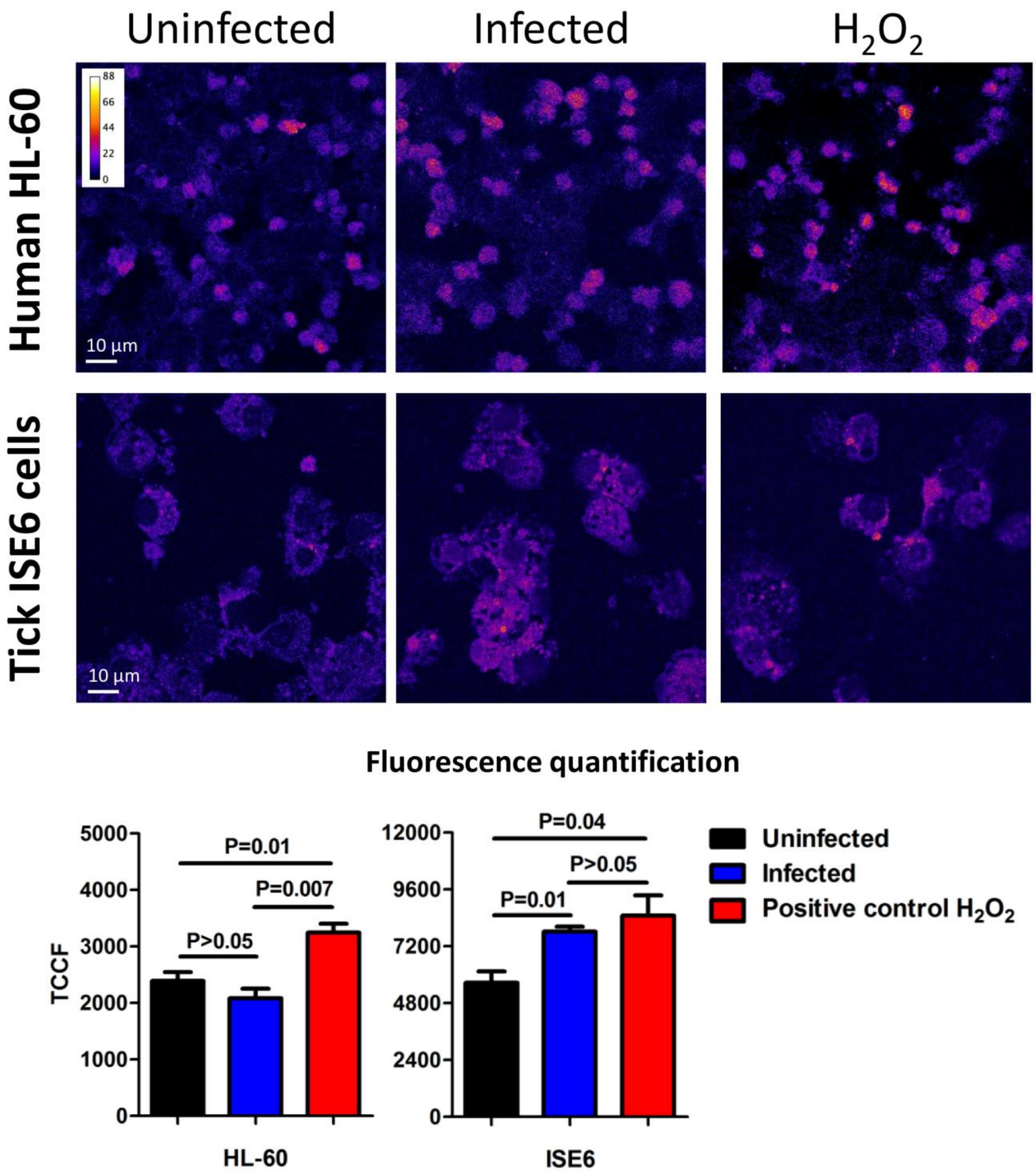

Supplementary Figure S1. Relative H_2O_2 concentration was determined with the cytoplasmic ratiometric reporter roGFP2-Orp1 by flow cytometry in Uninfected and *A. phagocytophilum*-infected ISE6 cells and HL-60 human cells differentiated to a neutrophil phenotype. Scale bar, 10 µm. Using ImageJ, an outline was drawn around each cell and area, mean fluorescence and integrated density were measured, along with several adjacent background readings. The total corrected cellular fluorescence (TCCF) = integrated density – (area of selected cell × mean fluorescence of background readings), was calculated.

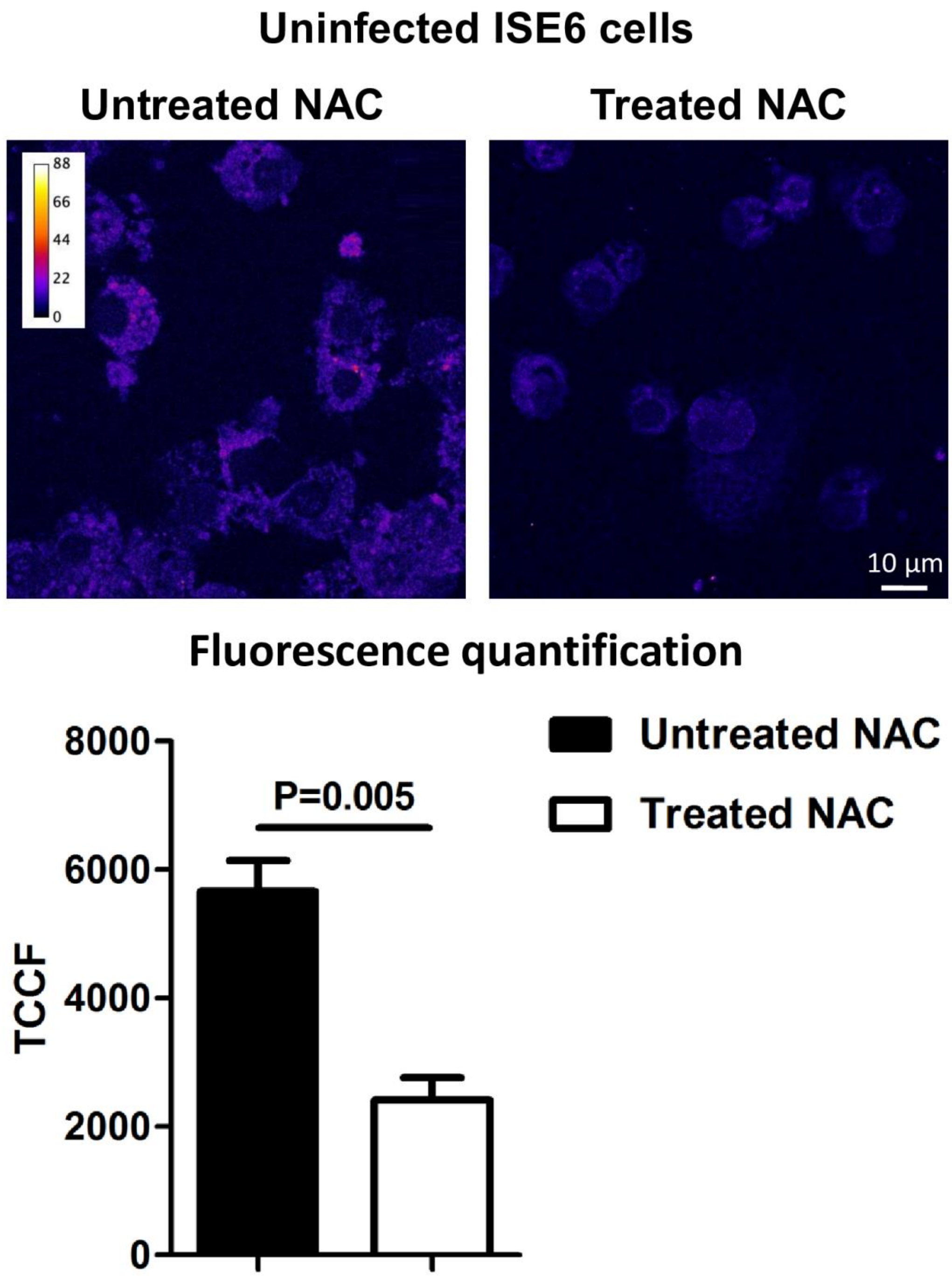

Supplementary Figure S2. Ratio images of ISE6 cells expressing roGFP2-Orp1 and treated with NAC. Uninfected ISE6 cells were treated for 2 days with 10 µM of the anti-oxidant N-acetyl cysteine (NAC). Scale bar, 10 µm. Using ImageJ, an outline was drawn around each cell and area, mean fluorescence and integrated density were measured, along with several adjacent background readings. The total corrected cellular fluorescence (TCCF) = integrated density – (area of selected cell × mean fluorescence of background readings), was calculated.

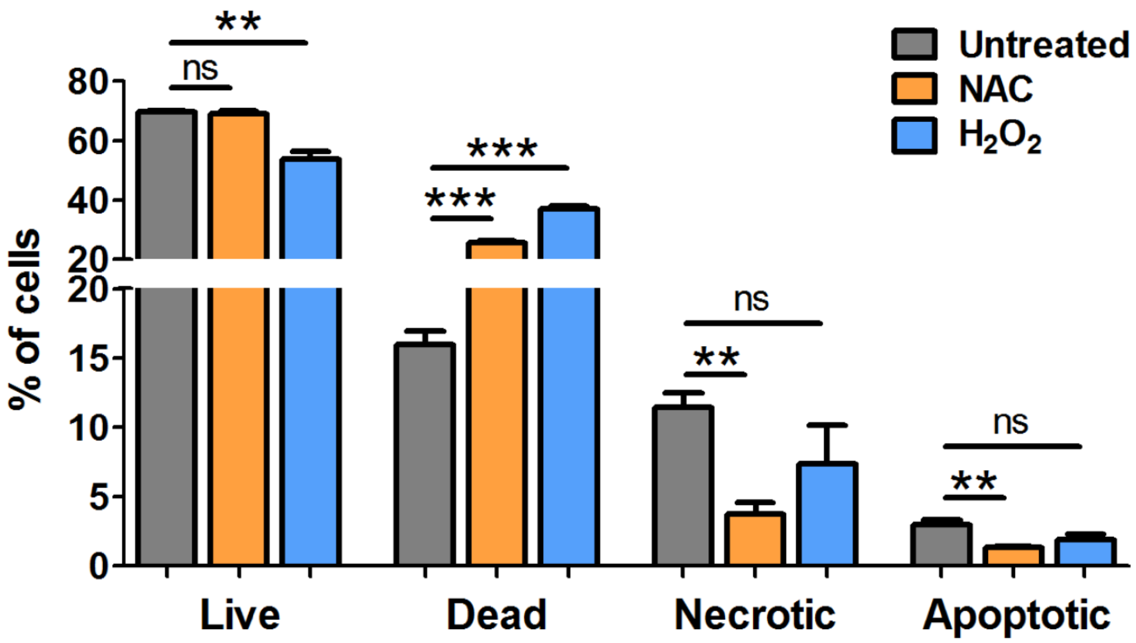

Supplementary Figure S3. Effect of treatment with N-acetyl cysteine (NAC) on the viability of ISE6 tick cells. The percentage of apoptotic, dead/late apoptotic, necrotic and viable cells was determined after 7 days in NAC-treated and untreated uninfected cells by flow cytometry after Annexin V-FITC and PI labeling, and the average of 4 replicated represented. H_2O_2 was used as control. Abbreviations: NAC, N-acetyl cysteine.

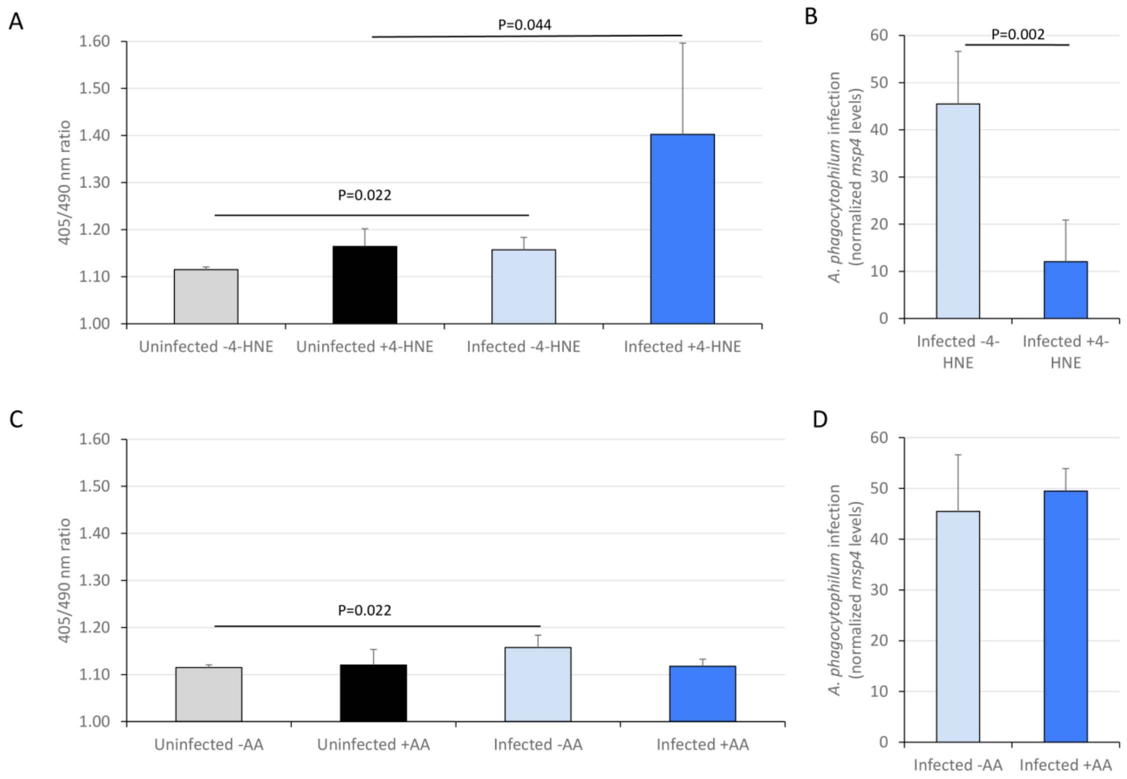

Supplementary Figure S4. Relative H_2O_2 concentration was determined with the cytoplasmic ratiometric reporter roGFP2-Orp1 by flow cytometry in ISE6 cells. Uninfected and infected cells were treated for 2 days with either 100 µM 4-Hydroxy-2-nonenal (A, B) or 2 µM Antimycin A (C, D). The *A. phagocytophilum* infection levels (B, D) were determined by *msp4* PCR normalizing against tick 16S rRNA. The ratios and normalized Ct values (Ave+S.D) were compared between groups by Student's t-test with unequal variance ($P<0.05$; N=4 biological replicates).

Supplementary Table S1. *A. phagocytophilum* Anka proteins identified in infected *I. scapularis* midgut, salivary glands and ISE6 cells.

Supplementary Table S2. Tick protein identification and quantitation.

Supplementary Table S3. Sequences of oligonucleotide primers used for real-time RT-PCR.
