# Supplementary material for: The redox metabolic pathways function to limit Anaplasma phagocytophilum infection and multiplication while preserving fitness in tick vector cells
Source: Sci Rep. 2019 Sep 13;9:13236. doi: 10.1038/s41598-019-49766-x (PMC6744499; doi:10.1038/s41598-019-49766-x)
Supplement: Supplementary file 4 — Supplementary Table S3 [file 41598_2019_49766_MOESM4_ESM.doc]

**Table S3.** Sequences of oligonucleotide primers used for real-time RT-PCR.

| **Gene** | **Genbank accession No.** | **Forward and Reverse primers (5’ – 3’)** |
| --- | --- | --- |
| *NADH-ubiquinone oxidoreductase, subunit NDUFB10/PDSW* | ISCW023078 | GTACTACTGGTACCACCGCC  TCCCTGAAGTCTTCGTGCAG |
| *ubiquinol cytochrome C reductase, subunit RIP1* | ISCW014071 | CATCCCAGGTAAAGCCGCTA  GGCTTCGTGGGATCACTTGT |
| *guanine nucleotide exchange factor for Rho and Rac GTPase* | ISCW013397 | GGCAAAGACCTGACGACGTA  GGTTCACCCTGGATGCTCTC |
| *thioredoxin reductase* | ISCW019121 | AGACACCGCAGTGGCTTAC  TGGATATTTGGGCCTCTCGC |
| *thioredoxin reductase* | ISCW000888 | TCGTGTTGGACAAGACTGGG  GGGTGGCTAAAGATCACCGT |
| *thioredoxin reductase* | ISCW007974 | GTCATGGTGCGGTCCATTTG  TCAAAGACGTCCTCCTGCAC |
| *glutaredoxin, GRX* | ISCW022680 | TGGTGTTCATGAAGGGGAGC CCAGACCACCTACGAGGGAT |
